# A quantitative projection of the net health effects of cannabis legalization in Germany

**DOI:** 10.1371/journal.pone.0330879

**Published:** 2025-09-02

**Authors:** Afschin Gandjour

**Affiliations:** Frankfurt School of Finance & Management, Frankfurt, Germany; University of Turin, ITALY

## Abstract

**Background/Aim:**

Cannabis consumption in Germany has been on the rise, culminating in the legalization of recreational cannabis in 2024. This shift aims to minimize the harms associated with black-market cannabis, such as exposure to contaminants, while regulating consumption to reduce health risks. The primary aim of this study is to quantitatively assess the net health effects of cannabis legalization in Germany by balancing harm reduction from fewer contaminants against potential risks from increased consumption.

**Methods:**

A quantitative projection model was employed to evaluate the potential net health effects of cannabis legalization in Germany. By estimating the likely increase in consumption and corresponding health risks, the study calculated quality-adjusted life year (QALY) losses due to cannabis use disorder (CUD) and long-term health impacts from both cannabis dependence and contamination exposure.

**Results:**

Projected increases in adult cannabis consumption may lead to 400,000–800,000 new users, resulting in approximately 2,300 additional cases of severe long-term mental health conditions. The corresponding QALY losses from CUD-related harms are estimated to be approximately nineteen times greater than the health gains from reduced contamination-related harm. Sensitivity analysis shows that consumption rates have a strong influence on net QALY outcomes, with even a 1% increase in cannabis use sufficient to produce net population-level harm.

**Conclusions:**

The findings suggest that cannabis legalization in Germany may not achieve the intended health benefits. Increased consumption, particularly among new users, may result in considerable public health burdens, with QALY losses associated with CUD outweighing gains from reduced contamination. Effective regulation and public health interventions are needed to minimize adverse health outcomes while avoiding a resurgence of black-market sales.

## Introduction

In recent years, cannabis consumption has become increasingly prevalent in Germany. Approximately 4.5 million adults aged 18–59 and around 340,000 adolescents aged 12–17 have used cannabis at least once in the past 12 months, whether regularly or occasionally. The proportion of people consuming cannabis within a 12-month period has been rising for over a decade [1]. This growing trend, along with public debates around health, safety, and economic considerations, has led to significant shifts in cannabis policy.

In 2024, Germany legalized cannabis for recreational use, having already allowed medical cannabis under strict conditions. This legalization marks a pivotal moment in the country’s drug policy, with broad social, legal, and economic implications. The decision reflects increasing societal acceptance of cannabis and a departure from the restrictive drug policies of the past. By legalizing recreational cannabis, Germany follows the lead of countries like Canada, Uruguay, and certain U.S. states.

According to the German government, previous drug policies had reached their limits. Despite prohibitions on possession and acquisition, cannabis consumption continued to rise, often linked to black-market sources that posed serious health risks. Contaminated cannabis, with unknown tetrahydrocannabinol (THC) content and potentially harmful additives or synthetic cannabinoids, exposed consumers to unpredictable dangers. In response, the new legislation aims to address these concerns by improving health protection, expanding education and prevention efforts, curbing organized drug crime, and strengthening protections for children and adolescents [2]. While the decriminalization of users is a consequence of the reform, it was not a major reason for the policy change; the focus lies instead on public health and safety.

Under the new legal framework, adults over the age of 18 can legally use and possess cannabis, including the right to grow up to three plants for personal use. Cannabis Social Clubs (CSCs) will allow adults to legally produce and consume cannabis in non-commercial community settings. However, cannabis use and possession remain illegal for minors under 18.

Although Germany’s regulatory framework includes strict measures to mitigate risks, evidence from the United States indicates that states with recreational marijuana laws (RMLs) experienced increases in adult (≥26 years) cannabis use and cannabis use disorder, with smaller or no changes among younger adults [3–5]. In Canada, where most provinces set the legal age at 18–19 (Quebec at 21) and where edibles/extracts were legalized nationally in October 2019 (except Quebec, which banned youth-friendly edibles and vaping products), larger increases in adolescent use were observed in provinces permitting these products compared with Quebec’s more restrictive regime [6].

Previous studies evaluating the health effects of cannabis legalization have largely focused on isolated outcomes—such as increases in cannabis use disorder [3], hospital admissions [7], or poisonings related to edibles [6]. However, few have attempted to synthesize these findings into an integrated health assessment. Moreover, to our knowledge, no prior study has quantitatively evaluated the trade-off between health benefits from reduced exposure to contaminated cannabis and the health risks from increased cannabis use following legalization. This study addresses that gap by projecting the net impact of legalization in Germany in terms of QALYs lost or gained. Against this backdrop, this study aims to:

1. Estimate the current burden of health harm—particularly acute intoxications and long-term mental health consequences—linked to contaminated cannabis in Germany.
2. Quantify the expected change in cannabis consumption and cannabis use disorder (CUD) following legalization.
3. Evaluate the net population-level health impact of legalizing cannabis without commercial retail access, by weighing reductions in contamination-related harm against increased use-related risks.

## Methods

### Long-term health impact of cannabis contamination in Germany

In Germany, the number of inpatient cases related to mental and behavioral disorders due to cannabinoid use was 19,091 in 2018, up from 3,392 in 2000, including 2,288 cases of acute intoxication [8]. Since hospital admission records do not differentiate between pure cannabis and contaminated or synthetic cannabinoid products, it is possible that cases involving contaminants are subsumed under cannabinoid-related diagnoses. While synthetic cannabinoids are far more prevalent in countries like the UK—where 35.7% of analytically confirmed intoxications with new psychoactive substances (NPS) were attributed to synthetic cannabinoids [9]—their use appears to be lower in Germany. Estimates suggest that 10% to 20% of cannabis products in Germany may be adulterated with synthetic cannabinoids [10]. On the other hand, most hospitalizations not classified as acute intoxications (e.g., psychotic episodes) are likely related to natural, high-potency cannabis. For instance, in Denmark, the incidence of cannabis-induced psychosis increased from 2.8 to 6.1 per 100,000 person-years between 2006 and 2016—an increase of 118%—primarily attributed to rising THC concentrations and increased use [11].

Assuming that all acute intoxication hospitalizations are due to contaminated or synthetic cannabis would overestimate their role. Conversely, assuming that none of the remaining hospitalizations involve contaminants would underestimate their contribution. As some degree of mutual cancellation occurs, adopting a range around the 2,288 acute intoxication cases—e.g., between 1,000 and 5,000—offers a conservative approximation that likely captures the true number of cases involving contaminated or synthetic cannabinoids.

It is therefore estimated that, in Germany, around 1,000–5,000 emergency cases and health issues annually may be caused by contaminated cannabis. Most health problems linked to contaminated cannabis are short-term, such as poisoning, respiratory issues, arrhythmias, or panic attacks, which are often treatable and do not result in long-term consequences [12]. However, in more severe cases, especially with chronic use of contaminated cannabis, lasting health damage may occur. This includes organ damage (e.g., kidneys or lungs), neurological disorders, and mental health conditions. Poisoning with heavy metals such as lead can also have long-term effects.

Given that most consumers exposed to contaminated cannabis experience only short-term symptoms [12], only a small fraction of the estimated 1,000–5,000 affected individuals per year in Germany are likely to suffer long-term health consequences. Based on U.S. poison center data, approximately 19% of cases with clinically significant outcomes (i.e., moderate or major effects requiring treatment or hospitalization) resulted in major adverse effects, defined as life-threatening symptoms or those leading to lasting disability [13]. Applying this proportion conservatively, we estimate that approximately 10% to 20% of hospitalized individuals could suffer permanent harm. This suggests that roughly 100–1,000 people per year in Germany may experience lasting health effects due to contaminated or synthetic cannabis.

Data from symptom inventories of synthetic cannabinoid users indicate that anxiety and depression symptoms are more prevalent and severe than psychosis-related symptoms [14]. Based on average scores from the Brief Symptom Inventory (BSI) reported in the study, anxiety and depression symptoms in this group appear to be approximately 40–50% more pronounced than psychotic symptoms.

It is often argued that the use of cannabis, particularly in contaminated forms, could increase the risk of users transitioning to harder drugs like cocaine or heroin. However, the evidence for this remains disputed, and there are no robust data supporting a direct link between contaminated cannabis and a significant number of hospital admissions due to a shift to harder drugs [15].

### Public health impacts of cannabis legalization

Across jurisdictions that have legalized non-medical cannabis, use has generally increased among legal-age adults, reflected in higher prevalence and more frequent or daily use, while youth trends have been more variable and context-dependent [16]. In Canada, for example, past-year cannabis use among adults (aged 15+) rose from approximately 15% in 2017 to 21% in 2019 [17], and to 26% in 2024 among individuals aged 16 and older using cannabis for non-medical purposes [18]. This upward trend coincided with an expanding legal market operating alongside the existing illicit one, increasing accessibility through diverse products and widespread retail channels.

Similar developments occurred in U.S. states such as Colorado, where the share of adults aged 26 and older reporting past-month use doubled between 2011 and 2014, after remaining stable from 2008 to 2011 [19]. These patterns suggest that legalization typically expands the overall market, with legal access supplementing rather than replacing illicit supply.

While public education campaigns have been implemented alongside legalization, their impact on curbing adult consumption appears limited. Nonetheless, variation across jurisdictions highlights the moderating role of regulatory design. For instance, Quebec—where the legal age is 21, and access to edibles and vaping products is restricted—has reported more stable consumption rates but higher uptake of legal versus illegal products.

The effects of legalization on youth consumption are less consistent. In Uruguay, legalization did not lead to increased adolescent cannabis use. In contrast, evidence from the United States and Canada is mixed. Longer follow-up studies suggest that while adolescent use may not rise immediately after legalization, increases over time cannot be ruled out [15].

High-quality controlled studies provide further insight. A U.S. twin study by Zellers et al. [20] found that adults living in states with legalized recreational cannabis used cannabis 24% more frequently than their genetically identical siblings in prohibition states—strong evidence that legalization itself contributes to increased use. Similarly, a controlled Canadian study of over 100,000 students found a 26% increase in youth cannabis use, particularly after the legalization of edibles [6].

While these studies reflect legalization contexts characterized by retail sales, product diversity, and commercial availability, Germany’s planned model is more restrictive, excluding legal sales and limiting access to cannabis clubs and home cultivation. As such, the projected increase in consumption under Germany’s framework is expected to be more limited—potentially in the range of 10–20% above baseline trends—driven by non-commercial access via social clubs and home cultivation.

### Health risks associated with cannabis use

It is estimated that individuals who use cannabis have about a 10% chance of developing CUD [21,22]. Among individuals with CUD, the lifetime risk of major depression is 22%, based on a hazard ratio (HR) of 1.84 [23] applied to the baseline population prevalence of MDD (12%; [24]). These figures suggest that roughly 10 percentage points of the MDD risk in individuals with CUD may be attributable to cannabis use. In addition, a meta-analysis suggests that the lifetime risk of developing a psychotic disorder among heavy cannabis users is approximately 4%, compared to about 1% in the general population [25]. This estimate is based on a consistent increase in the risk of psychosis-related outcomes with higher levels of cannabis exposure across all included studies [25]. Due to ethical concerns, including potential harm and challenges with informed consent, randomized controlled trials to establish causality are infeasible. Therefore, among individuals who experience long-term health damage due to cannabis dependence, approximately 30% may suffer from psychotic disorders. While long-term health damage is more prevalent among individuals with cannabis dependence, non-dependent users may also be affected, particularly with high-potency products.

Thus, it is assumed that approximately 1–2% of new cannabis users may develop long-term mental health problems, such as depression or psychosis, with elevated risks among those with genetic vulnerability, early onset of use, or exposure to high-THC cannabis.

In terms of annual hospitalizations resulting from cannabinoid use, data indicate 19,091 hospitalizations in 2018 [8] among an estimated 4 million past-year cannabis users, resulting in an annual hospitalization rate of about 0.5%.

### The impact of cannabis legalization on hospital admissions

Studies do not show a clear decline in hospital admissions among adults following cannabis legalization, and in many cases, an increase has been observed. According to a systematic review conducted by Manthey et al. [15], most of the studies on CUD in the healthcare sector used simple pre-post analyses and interrupted time series analyses. When limiting the analysis of CUD to studies that included data from control regions (i.e., states where cannabis had not been legalized or regions within the same state without cannabis retailers), three studies reported an increase in hospitalizations due to CUD.

A systematic review by Athanassiou et al. [5] yielded similar results, analyzing four studies on the impact of RMLs on healthcare service use, including cannabis-related hospitalizations, emergency department visits, and reported cannabis exposures. Three of the studies reported an increase in healthcare service utilization linked to RML status, while one study found a decrease in cannabis-related treatment admissions among young adults in seven states where cannabis was legalized.

In a study published in October 2023 [7], which was conducted after the searches for the above reviews, legalization without commercialization in Canada was associated with a gradual monthly decrease in cannabis-related hospitalizations of 0.06 cases per 100,000 individuals (95% CI: −0.08 to −0.03). However, commercialization and the COVID-19 pandemic were linked to an immediate increase of 0.83 (95% CI, 0.30 to 1.30) hospitalizations per 100,000 individuals. The initial decrease in hospitalizations following legalization may have resulted from reduced cannabis availability in the early stages, due to national shortages of legal cannabis products and a contraction of the illegal market, evidenced by increased enforcement activities and the voluntary closure of illicit dispensaries during the transition to a legal market [7].

Without the legalization of edibles, a moderate increase in hospital admissions related to other forms of cannabis (e.g., flowers) is likely, particularly among inexperienced users or those using highly concentrated products. However, this increase is expected to be smaller compared to countries where edibles are legal. The sale of cannabis edibles could lead to a higher rate of medically treated intoxications in children [15]. Intoxications among adults may also rise, though likely only temporarily [15].

If hospital admissions remain constant after legalization, it can be hypothesized that the positive effects of reduced contamination and improved product safety may offset the potential negative consequences of increased consumption.

### Assessing QALY losses

To compare the potential short- and long-term harm experienced by new cannabis users with the harm caused by avoidable contamination, calculating quality-adjusted life years (QALYs) can be useful. QALYs assess the health benefits (or detriments) of a specific health condition by considering both the quality of life and the years lived.

The total QALY loss is calculated using the formula:


Total QALY Loss = N · Q · D,

where N represents the number of cases (e.g., individuals experiencing harm), Q denotes the overall reduction in quality of life for each case during the harm period (regardless of the time unit), and D is the duration (the length of time the harm persists, in years or fractions of a year).

When new users suffer long-term harm, the resulting impact on quality of life must be considered. For instance, chronic conditions such as depression or anxiety disorders can significantly reduce both quality of life and the number of healthy life years, even in the absence of other comorbidities. Severe mental health disorders, including schizophrenia or psychosis, may result in an annual QALY decrement of 0.4 to 0.7, depending on severity [26]. Assuming a discounted remaining life expectancy of 30–35 years—based on an undiscounted average of 50–60 years for young adults—this corresponds to a total discounted QALY loss of approximately 10–20 QALYs per affected individual.

For individuals who develop non-severe long-term mental health issues, such as anxiety disorders, depression, or cognitive impairments due to cannabis dependence, the QALY loss varies based on the severity of the conditions. Studies show that the annual QALY loss for individuals with anxiety disorders or depression can range from 0.1 to 0.3 QALYs per year, depending on symptom severity [27].

Long-term damage from cannabis contamination can range from severe consequences such as schizophrenia to persistent but milder conditions like anxiety disorders or depression [12,28]. In addition, serious health consequences from contaminants—such as lung damage (from pesticides or synthetic substances), organ damage (affecting the liver or kidneys), or neurological injury due to toxic chemicals—have also been reported [12]. The latter outcomes can lead to significant and often permanent reductions in quality of life and may result in QALY losses estimated at 0.25 to 0.45 per year [29–31].

For more mild but persistent health effects, such as anxiety disorders, mild respiratory diseases, or allergic reactions, the QALY loss would be similar to that of non-severe cannabis dependence.

Regular cannabis use is primarily associated with long-term mental and cognitive effects. However, it can also lead to acute intoxications requiring short hospital stays, typically involving symptoms such as severe nausea, vomiting, confusion, or breathing difficulties. For such cases, the temporary quality-of-life reduction is estimated at 20–30% (i.e., 0.2 to 0.3 QALYs/year). Applied over a symptomatic period of 3–7 days, this corresponds to a QALY loss of approximately 0.004 per episode (range: 0.002–0.006) for cannabis-related intoxications not involving contaminants.

By contrast, contaminated black-market cannabis poses more immediate physical risks due to exposure to toxic or chemical substances. These risks can lead to more severe and prolonged intoxications—particularly those involving synthetic cannabinoids—which may result in poisoning, respiratory distress, or, in some cases, lasting organ damage. For moderate contaminant- or synthetic cannabinoid-related intoxications, the estimated QALY loss is approximately 0.010 per episode (range: 0.006–0.015) [32], reflecting greater symptom severity and longer recovery durations. These estimates reflect only the short-term disutility associated with hospitalization and do not account for potential long-term consequences such as permanent organ or neurological damage.

### Sensitivity analysis

In a univariate sensitivity analysis, a tornado diagram was employed to visually represent the impact of varying key parameters on the model’s outcomes. Each parameter was independently adjusted within its plausible range, while all other variables remained constant at their baseline values. This approach allowed us to identify the most influential parameters on the QALY loss ratio between CUD and contamination.

## Results

Assuming conservatively that approximately 4 million people in Germany use cannabis, either regularly or occasionally [1], the projected increase in use following legalization can be estimated. A 10% increase would correspond to approximately 400,000 additional users, while a 20% increase would result in around 800,000 new users.

Assuming that 1–2% of new cannabis users develop long-term mental health disorders, and that approximately 30% of these cases involve severe, lasting harm (such as psychosis or schizophrenia), this would correspond to approximately 2,300 individuals developing serious mental health conditions. Consequently, the total QALY loss could range from 23,000–47,000 QALYs, based on an estimated loss of 10–20 QALYs per person.

As shown in Table 1, the predicted combined short- and long-term harm from new cases of cannabis dependence is approximately 19 times higher compared to harm caused by contamination, leading to a net increase in QALY loss. The primary reason for this difference is severe long-term harm from new users.

**Table 1:** Population-wide quality-adjusted life year loss by type of harm.

	Severe long-term harm	Non-severe long-term harm	Short-term harm	Sum
Contamination	2,041	1,480	25.0	3,545
CUD	35,100	32,760	11.5	67,871

CUD: cannabis use disorder.

**Fig 1:**
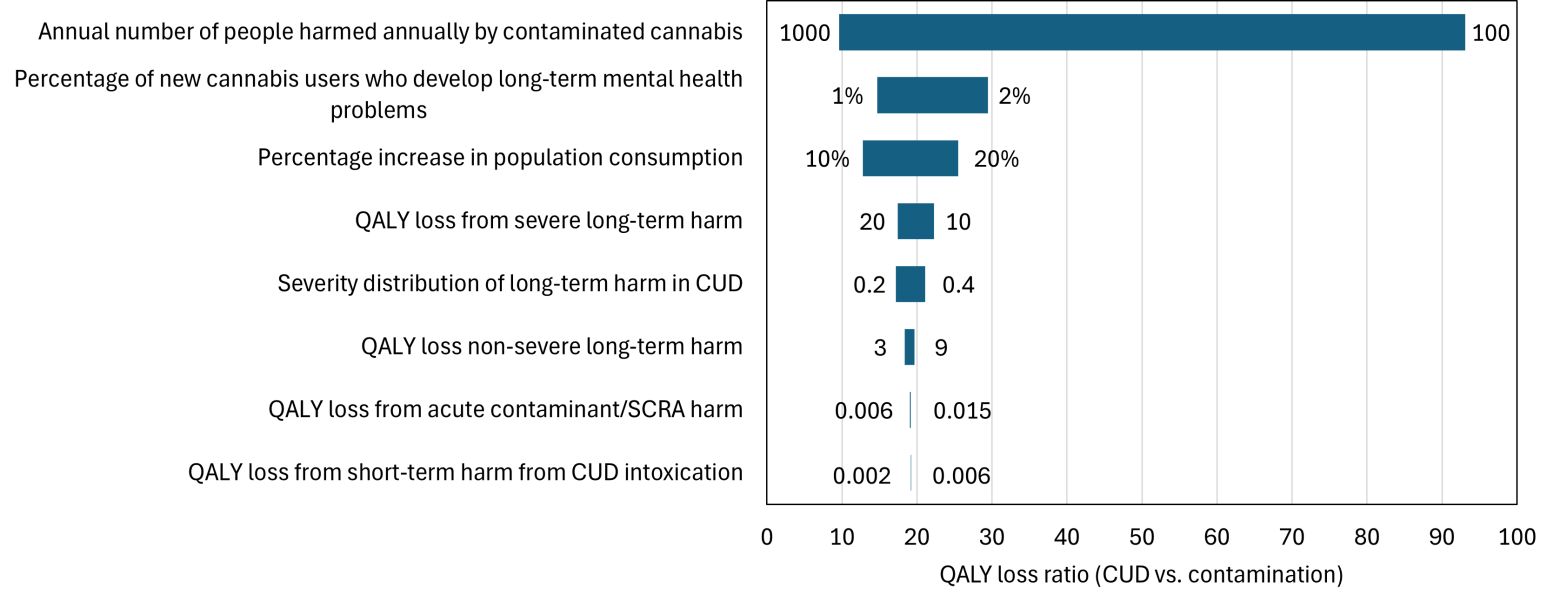
One-way sensitivity analysis on the quality-adjusted life year (QALY) loss ratio (cannabis use disorder (CUD) vs. contamination). Variables are ordered by their impact on the QALY loss ratio. SCRA, synthetic cannabinoid receptor agonist.

In all sensitivity analyses, the QALY loss from new cases of CUD exceeds the QALY gain from avoided contamination-related harms. The variable with the greatest influence on the QALY loss ratio between CUD and contamination is the annual number of individuals harmed by contaminated cannabis (Fig 1). A threshold sensitivity analysis indicates that even a 1% increase in cannabis consumption would be sufficient to result in net population-level harm.

While the number of short-term hospitalizations due to contamination is comparable to that of CUD-related hospitalizations, the number of hospitalizations resulting from long-term harm is markedly lower (Table 2). Notably, the calculation does not account for repeated hospitalizations associated with long-term harm, which would further widen the difference.

**Table 2:** Population-wide annual number of hospitalizations by type of harm.

	Severe long-term harm	Short-term harm	Sum
Contamination	500	2,500	3,000
CUD	2,340	2,864	5,204

CUD: cannabis use disorder.

## Discussion

### Principal findings

This study suggests that in Germany, the projected long-term QALY losses—primarily driven by an increased incidence of mental health disorders and hospitalizations associated with CUD—outweigh the potential QALY gains from reducing cannabis contamination, such as fewer cases of acute intoxications and related hospitalizations. The anticipated rise in adult consumption among new and existing users could lead to significant public health burdens, especially if this increase is linked to a higher prevalence of mental health disorders.

Furthermore, the study projects that hospitalization rates will not decline following cannabis legalization, a finding consistent with international data. Notably, this study appears to be the first to quantitatively demonstrate the trade-off between an increase in CUD-related hospitalizations and a decrease in contamination-related hospitalizations. It highlights that, despite relatively similar rates of additional hospitalization for new CUD cases and avoided hospitalizations from contaminations, at least in the short term, the projected QALY loss from new CUD appears to be substantially larger than the QALY gain from avoided contaminations in the long run.

### Study limitations

This analysis has several limitations that warrant consideration. First, the model does not account for regional differences in consumption trends or enforcement, which may lead to varying impacts across Germany. Second, while the analysis estimates QALY losses from both cannabis dependence and contamination, it relies on assumptions regarding the probability and severity of harm, which may not fully capture the complexities of real-world health outcomes. Third, the study does not examine broader societal effects, such as potential changes in crime rates or economic impacts from job creation in the cannabis industry, which could influence the overall assessment of legalization’s effects. Fourth, the analysis does not incorporate potential increases in traffic-related harms following legalization. Several studies have reported a link between cannabis legalization and rising numbers of traffic-related incidents, including fatal accidents. However, none of the 14 studies included in a recent review found a decrease in traffic safety indicators [15]. Finally, the analysis did not consider cannabis as a gateway drug, as there is no conclusive scientific evidence that cannabis itself functions as such. While cannabis users may be more likely to try other drugs, this could be due to external factors such as environment, availability, and personal predispositions. Therefore, the gateway drug theory is more likely based on correlation than clear causation.

### Scientific and operational perspectives

From a scientific standpoint, this study contributes a novel quantitative framework for balancing the health risks and benefits of cannabis legalization—filling a critical gap in the existing literature that often focuses on isolated outcomes. By applying a QALY-based approach, the study integrates diverse health consequences (e.g., mental health disorders, hospitalizations, contaminant exposure) into a unified metric, enabling clearer comparisons and policy-relevant insights.

Operationally, the findings highlight the need for robust post-legalization surveillance systems to monitor cannabis use, hospital admissions, and mental health trends. The study also underscores the importance of anticipatory regulatory strategies—such as THC potency limits, targeted public health campaigns, and pricing mechanisms—to mitigate excessive consumption while maintaining the attractiveness of the legal market.

### Policy trade-offs and future challenges

While this analysis is based on Germany’s current legal framework, it does not consider the potential introduction of cannabis store sales, which are not part of the existing legislation. Without retail outlets, it will be challenging to fully eradicate the black market. On the other hand, the introduction of store sales could treat cannabis as a commercial product, potentially leading to advertising and promotion, which might increase consumption—unlike the non-commercial nature of CSCs, which focus on personal use.

Countries like Canada have experienced an increase in consumption, particularly among recreational users, due to the regular sale of cannabis in stores. In September 2019, during Canada’s first year of legalization, legal recreational market shares across the 10 provinces varied from 13% to 70%, considering commercial store sales [33]. A non-commercial model minimizes the risk of excessive advertising and prevents cannabis from being treated like a standard consumer product, promoting moderate, controlled consumption without commercial incentives to increase use.

There is a clear conflict between the goal of reducing the black market and the risk of promoting more consumption. The challenge for policymakers is to find a balance between these objectives by regulating the market effectively without unnecessarily increasing consumption [15]. As suggested by the findings of the present study, efforts to fully eliminate the black market through comprehensive legalization may come at the expense of public health—namely, through increased consumption and related harms. In other words, the eradication of the black market may be partly incompatible with health protection objectives. To minimize negative impacts, legal cannabis must be attractive enough to persuade existing users to switch from illegal sources but not so attractive as to encourage first-time use. However, achieving both objectives equally may not be possible in practice due to many regulatory challenges [15].

Even if the health effects of legalization tend to be negative, as suggested by this study, other positive effects of legalization, although not the primary motivation, may still exist. The introduction of CSCs in Germany could ease the burden on the judiciary, as fewer people would be prosecuted for possession or use of cannabis. However, police concerns that legalization will not lead to operational relief stem from anticipated additional responsibilities, including enforcing new regulations, continuing to monitor illegal cannabis activity, and ensuring road safety. While minor offenses may decline, overseeing the legal market and addressing new forms of illegal trade—such as unauthorized distribution from CSCs or diversion to non-members—could pose significant enforcement challenges.

Based on the available evidence, it is unlikely that cannabis legalization in Germany will reduce violent or property crime, nor is it expected to lead to an increase in criminal activity in the short term [15]. Nevertheless, CSCs do create jobs, mainly in cultivation, care, administration, and legal consultation. However, while the employment opportunities generated by CSCs are beneficial, they alone do not seem sufficient to justify the comprehensive cannabis legalization package in Germany.

This study highlights another dilemma: the individual consumer’s preference for uncomplicated access does not align with societal preferences, as the latter are unlikely to be satisfied due to higher rates of CUD and the associated QALY loss. The individual consumer’s desire for easy access to cannabis does not consider the societal harm caused by increased use, such as health risks, addiction, or higher healthcare costs. These societal harms are negative externalities, meaning they are costs imposed on others or society that are not reflected in the private decisions of the individual consumer. Policymakers must account for this misalignment. Pigouvian taxes—taxes levied on activities that generate negative externalities—are designed to correct this market failure by increasing the private cost of consumption to reflect its full social cost. Alternatively, other regulatory interventions may be used to internalize these externalities, ensuring that private decisions better align with the broader interests of society.

While Pigouvian taxes can help address the negative externalities of cannabis consumption, setting the tax rate too high risks undermining the legal market by driving consumers back to the black market. The solution lies in finding a balance between discouraging excessive use and keeping legal cannabis affordable compared to illegal alternatives.

### Conclusion

In conclusion, based on our projections, the legalization of cannabis in Germany may not deliver the harm reduction benefits envisioned by the government. While improved access to recreational cannabis may be seen as a primary benefit, prioritizing consumer enjoyment is a controversial objective, as easier access and increased use may lead to negative health consequences.

To mitigate the public health risks associated with increased cannabis consumption, policymakers must strike a careful balance in regulation. Effective strategies may include regulating THC potency, implementing public health campaigns to educate users, and applying targeted taxation to discourage overuse. However, it is crucial to ensure that such measures do not drive consumers back to the black market through excessive taxation or over-regulation.

## References

[1] Der Beauftragte der Bundesregierung für Sucht- und Drogenfragen. Cannabiskonsum in Deutschland. 2023. https://datenportal.bundesdrogenbeauftragter.de/cannabis#:~:text=Der%20Cannabiskonsum%20der%2018%2D%20bis,den%20vergangenen%2012%20Monaten%20Cannabis

[2] Bundesministerium für Gesundheit. Fragen und Antworten zum Cannabisgesetz. 2024. https://www.bundesgesundheitsministerium.de/themen/cannabis/faq-cannabisgesetz.html

[3] Cerdá M, Mauro C, Hamilton A, Levy NS, Santaella-Tenorio J, Hasin D, et al. Association between recreational marijuana legalization in the United States and changes in marijuana use and cannabis use disorder from 2008 to 2016. JAMA Psychiatry. 2020;77(2):165–71. 31722000 10.1001/jamapsychiatry.2019.3254 PMC6865220

[4] Kim JH, Weinberger AH, Zhu J, Barrington-Trimis J, Wyka K, Goodwin RD. Impact of state-level cannabis legalization on poly use of alcohol and cannabis in the United States, 2004-2017. Drug Alcohol Depend. 2021;218:108364. doi: 10.1016/j.drugalcdep.2020.108364 33143941

[5] Athanassiou M, Dumais A, Zouaoui I, Potvin S. The clouded debate: A systematic review of comparative longitudinal studies examining the impact of recreational cannabis legalization on key public health outcomes. Front Psychiatry. 2023;13:1060656. doi: 10.3389/fpsyt.2023.1060656 36713920 PMC9874703

[6] Mital S, Nguyen HV. Legalizing Youth-Friendly Cannabis Edibles and Extracts and Adolescent Cannabis Use. JAMA Netw Open. 2025;8(4):e255819. doi: 10.1001/jamanetworkopen.2025.5819 40249613 PMC12008758

[7] Myran DT, Gaudreault A, Konikoff L, Talarico R, Liccardo Pacula R. Changes in Cannabis-Attributable Hospitalizations Following Nonmedical Cannabis Legalization in Canada. JAMA Netw Open. 2023;6(10):e2336113. doi: 10.1001/jamanetworkopen.2023.36113 37796504 PMC10556968

[8] Gahr M, Ziller J, Keller F, Muche R, Preuss UW, Schönfeldt-Lecuona C. Incidence of inpatient cases with mental disorders due to use of cannabinoids in Germany: a nationwide evaluation. Eur J Public Health. 2022;32(2):239–45. 35043164 10.1093/eurpub/ckab207 PMC8975525

[9] Craft S, Dunn M, Vidler D, Officer J, Blagbrough IS, Pudney CR, et al. Trends in hospital presentations following analytically confirmed synthetic cannabinoid receptor agonist exposure before and after implementation of the 2016 UK Psychoactive Substances Act. Addiction. 2022;117(11):2899–906. doi: 10.1111/add.15967 35665553 PMC9796520

[10] Geyer T. Chemisch gestrecktes Cannabis: So groß ist das Problem in Deutschland. 2021. https://www.vice.com/de/article/chemisch-gestrecktes-cannabis-so-gross-ist-das-problem-in-deutschland/

[11] Hjorthøj C, Larsen MO, Starzer MSK, Nordentoft M. Annual incidence of cannabis-induced psychosis, other substance-induced psychoses and dually diagnosed schizophrenia and cannabis use disorder in Denmark from 1994 to 2016. Psychol Med. 2021;51(4):617–22. doi: 10.1017/S0033291719003532 31839011

[12] Maine Office of Cannabis Policy. Toxicity and Health Impacts of Cannabis Contaminants. 2022. https://www.maine.gov/dafs/ocp/sites/maine.gov.dafs.ocp/files/inline-files/Toxicity%20and%20Health%20Impacts%20of%20Cannabis%20Contaminants.pdf

[13] Law R, Schier J, Martin C, Chang A, Wolkin A. Notes from the Field: Increase in Reported Adverse Health Effects Related to Synthetic Cannabinoid Use - United States, January-May 2015. MMWR Morb Mortal Wkly Rep. 2015;64(22):618–9. 26068566 PMC4584925

[14] Mensen VT, Vreeker A, Nordgren J, Atkinson A, de la Torre R, Farré M, et al. Psychopathological symptoms associated with synthetic cannabinoid use: a comparison with natural cannabis. Psychopharmacology (Berl). 2019;236(9):2677–85. doi: 10.1007/s00213-019-05238-8 30968175 PMC6695363

[15] Manthey J, Hayer T, Jacobsen B, Kalke J, Klinger S, Rehm J, et al. Technischer Bericht. Auswirkungen der Legalisierung von Cannabis. Hamburg, Deutschland: Institut für interdisziplinäre Sucht- und Drogenforschung (ISD). 2023.

[16] Mekonen Yimer T, Hoch E, Fischer B, Dawson D, Hall W. The adverse public health effects of non-medical cannabis legalisation in Canada and the USA. Lancet Public Health. 2025;10(2):e148–59. doi: 10.1016/S2468-2667(24)00299-8 39909688

[17] Statistics Canada. Research to insights: Cannabis in Canada. 2023. https://www150.statcan.gc.ca/n1/pub/11-631-x/11-631-x2023006-eng.htm

[18] Health Canada. Canadian Cannabis Survey 2024: Summary. 2024. https://www.canada.ca/en/health-canada/services/drugs-medication/cannabis/research-data/canadian-cannabis-survey-2024-summary.html

[19] Roberts BA. Legalized Cannabis in Colorado Emergency Departments: A Cautionary Review of Negative Health and Safety Effects. West J Emerg Med. 2019;20(4):557–72. doi: 10.5811/westjem.2019.4.39935 31316694 PMC6625695

[20] Zellers SM, Ross JM, Saunders GRB, Ellingson JM, Anderson JE, Corley RP, et al. Impacts of recreational cannabis legalization on cannabis use: a longitudinal discordant twin study. Addiction. 2023;118(1):110–8. doi: 10.1111/add.16016 36002928 PMC10086942

[21] Lopez-Quintero C, Pérez de los Cobos J, Hasin DS, Okuda M, Wang S, Grant BF, et al. Probability and predictors of transition from first use to dependence on nicotine, alcohol, cannabis, and cocaine: results of the National Epidemiologic Survey on Alcohol and Related Conditions (NESARC). Drug Alcohol Depend. 2011;115(1–2):120–30. doi: 10.1016/j.drugalcdep.2010.11.004 21145178 PMC3069146

[22] Centers for Disease Control and Prevention. Understanding Your Risk for Cannabis Use Disorder. 2024. https://www.cdc.gov/cannabis/health-effects/cannabis-use-disorder.html

[23] Jefsen OH, Erlangsen A, Nordentoft M, Hjorthøj C. Cannabis Use Disorder and Subsequent Risk of Psychotic and Nonpsychotic Unipolar Depression and Bipolar Disorder. JAMA Psychiatry. 2023;80(8):803–10. doi: 10.1001/jamapsychiatry.2023.1256 37223912 PMC10209828

[24] Bains N, Abdijadid S. Major Depressive Disorder. StatPearls. Treasure Island (FL): StatPearls Publishing. 2025. 32644504

[25] Marconi A, Di Forti M, Lewis CM, Murray RM, Vassos E. Meta-analysis of the Association Between the Level of Cannabis Use and Risk of Psychosis. Schizophr Bull. 2016;42(5):1262–9. doi: 10.1093/schbul/sbw003 26884547 PMC4988731

[26] Aceituno D, Pennington M, Iruretagoyena B, Prina AM, McCrone P. Health State Utility Values in Schizophrenia: A Systematic Review and Meta-Analysis. Value Health. 2020;23(9):1256–67. doi: 10.1016/j.jval.2020.05.014 32940244

[27] Roberts J, Lenton P, Keetharuth AD, Brazier J. Quality of life impact of mental health conditions in England: results from the adult psychiatric morbidity surveys. Health Qual Life Outcomes. 2014;12:6. 24422899 10.1186/1477-7525-12-6 PMC3901021

[28] Radhakrishnan R, Wilkinson ST, D’Souza DC. Gone to Pot - A Review of the Association between Cannabis and Psychosis. Front Psychiatry. 2014;5:54. doi: 10.3389/fpsyt.2014.00054 24904437 PMC4033190

[29] Cooper JT, Lloyd A, Sanchez JJG, Sörstadius E, Briggs A, McFarlane P. Health related quality of life utility weights for economic evaluation through different stages of chronic kidney disease: a systematic literature review. Health Qual Life Outcomes. 2020;18(1):310. doi: 10.1186/s12955-020-01559-x 32957990 PMC7507735

[30] Hui Y, Wang H, Guo G, Yang W, Zhang X, Yang J, et al. Association Between Quality of Life Defined by EuroQol Group 5 Dimension and Composite Inferior Outcome Among Inpatients with Cirrhosis. Clinical Interventions in Aging. 2024;19:551–60. 38528882 10.2147/CIA.S444842 PMC10962662

[31] Salant N, Mohiuddin S, Zhang Y, Ayiku L, Lokuge K, Jacklin P, et al. EQ-5D Based Utility Values for Adults with Chronic Obstructive Pulmonary Disease: A Systematic Review, Meta-Analysis, and Meta-Regression. COPD. 2024;21(1):2385358. doi: 10.1080/15412555.2024.2385358 39081103

[32] Waters ML, Dargan PI, Yates C, Dines AM, Eyer F, Giraudon I, et al. Clinical effects of cannabis compared to synthetic cannabinoid receptor agonists (SCRAs): a retrospective cohort study of presentations with acute toxicity to European hospitals between 2013 and 2020. Clin Toxicol (Phila). 2024;62(6):378–84. 38934347 10.1080/15563650.2024.2346125

[33] Armstrong MJ. Legal cannabis market shares during Canada’s first year of recreational legalisation. Int J Drug Policy. 2021;88:103028. doi: 10.1016/j.drugpo.2020.103028 33221614

